# The Default Mode Network and Altered Consciousness in Epilepsy

**DOI:** 10.3233/BEN-2011-0310

**Published:** 2011-03-29

**Authors:** Nathan B. Danielson, Jennifer N. Guo, Hal Blumenfeld

**Affiliations:** ^1^Department of NeurologyYale University School of Medicine333 Cedar StreetNew HavenCTUSA; ^2^Department of NeurobiologyYale University School of Medicine333 Cedar StreetNew HavenCTUSA; ^3^Department of NeurosurgeryYale University School of Medicine333 Cedar StreetNew HavenCTUSA

**Keywords:** Epilepsy, consciousness, default mode network

## Abstract

The default mode network has been hypothesized based on the observation that specific regions of the brain are consistently activated during the resting state and deactivated during engagement with task. The primary nodes of this network, which typically include the precuneus/posterior cingulate, the medial frontal and lateral parietal cortices, are thought to be involved in introspective and social cognitive functions. Interestingly, this same network has been shown to be selectively impaired during epileptic seizures associated with loss of consciousness. Using a wide range of neuroimaging and electrophysiological modalities, decreased activity in the default mode network has been confirmed during complex partial, generalized tonic-clonic, and absence seizures. In this review we will discuss these three seizure types and will focus on possible mechanisms by which decreased default mode network activity occurs. Although the specific mechanisms of onset and propagation differ considerably across these seizure types, we propose that the resulting loss of consciousness in all three types of seizures is due to active inhibition of subcortical arousal systems that normally maintain default mode network activity in the awake state. Further, we suggest that these findings support a general “network inhibition hypothesis”, by which active inhibition of arousal systems by seizures in certain cortical regions leads to cortical deactivation in other cortical areas. This may represent a push-pull mechanism similar to that seen operating between cortical networks under normal conditions.

## 1. Introduction

Epileptic seizures can vary widely in terms of onset, propagation, and clinical manifestation, and often dramatically impair the patient's state of consciousness. The associated cognitive deficits typically occur during seizure activity and persist even after the seizure's end. Interestingly, the specific nature and degree of these deficits can vary widely across patients and even across seizures. For example, in absence seizures, it has been proposed that the variable impairments observed are likely the result of focal disruptions of specific brain networks [12,38]. This view has been supported by mounting evidence that even so-called "generalized" seizures, such as absence and tonic-clonic seizures, are of focal origin and likely do not affect the whole brain homogeneously [4,20,68]. Thus, networks affected by generalized seizures may in some ways resemble those involved in partial seizures [10].

Studies of different seizure types have revealed highly variable and complex changes in metabolism and blood flow during these events. While different seizure types vary widely in terms of initiation and propagation, all have been associated with widespread deactivations in the late ictal and post-ictal periods. Remarkably, many of the deactivations observed during seizures coincide with the architecture of an independently established network, the so-called default mode network (DMN). The anatomy of the DMN is consistently activated at rest and deactivated during periods of goal-directed behavior. This seemingly paradoxical reduced activity of the DMN during epileptic seizures might be related the behavioral correlates of these events. However, a precise understanding of the role and the mecha-

ISSN 0953-4180/11/$27.50 © 2011 – IOS Press and the authors. All rights reserved nisms by which activity in this network is reduced have yet to be established.

Due to the reproducibility and transient nature of seizures, epilepsy is an excellent model for studies of consciousness. While consciousness has been a historically difficult concept to define, Plum and Posner have distinguished between the level of consciousness versus the content of consciousness, which are conceptually distinct and may be subserved by different neural substrates [58]. We have recently summarized the specialized networks regulating the level of consciousness, which we refer to as the "consciousness system" in analogy to sensory, motor and other systems [13, 15]. The consciousness system includes subcortical structures important for arousal as well as medial and lateral regions of higher order association cortex, overlapping substantially with the DMN. With regard to the functions of the default mode, it is crucial to distinguish external awareness and self-awareness. External awareness can be thought of as responses to environmental stimuli, whereas self-awareness is a contained process requiring no external input. This distinction is important given the hypothesized function of the DMN in self-awareness [16,60].

In this review, we will first present a general overview of the DMN and its constituent structures and proposed functions. Next we will discuss complex-partial, tonic-clonic, and absence seizures and will summarize the evidence for DMN involvement during these events. We propose that loss of consciousness in seizures is caused by abnormal activity in subcortical and cortical regions including the default mode network.

## 2. The default mode network

The default mode network (DMN) was hypothesized following observations that specific areas of the brain are consistently activated at rest (either passive viewing or lying with eyes closed) and deactivated in goal-directed behaviors [60]. The default mode areas are commonly thought to include the posterior cingulate cortex / precuneus in the medial parietal lobe, the medial prefrontal cortex, and the lateral parietal cortex [50,60]. The default mode network has also emerged through resting functional connectivity analysis, which shows the regions of the DMN are significantly positively correlated with each other and anti-correlated with a network involving numerous attention-related structures (Fig. 1).

Functionally, the DMN has been thought to govern spontaneous, stimulus-independent self-referential and social cognitive functions [48]. Intrinsic activity in the DMN may also be a necessary online function to direct incoming environmental stimuli according to their appropriate emotional salience or attentional priorities. Functional connectivity analyses have also identified the DMN using correlated spontaneous slow-wave fluctuations in the resting condition [29]. However, similar analyses have also identified the DMN in the anesthetized state, coma, and slow-wave sleep [44, 69]. Because functional connectivity is preserved even in these states, it seems spontaneous fluctuations are not in themselves sufficient for maintenance of the conscious state. Furthermore, the idea of a "default mode" of brain function remains controversial, partially because of the difficulties of defining such an unconstrained state as default [54]. However, the idea of a "default" brain state is particularly interesting given the enormous amount of energy the brain consumes at baseline without apparent stimulation by environmental input [50,64]. While neuroimaging studies using differencing methods have traditionally been designed around the assumption that the brain is driven by momentary environmental sensory inputs, insights from magnetic resonance spectroscopy (MRS) and positron emission tomography (PET) studies showing high levels of energy consumed by the brain even in the absence of explicit sensorimotor tasks have challenged this view and have demonstrated the potential value of studying a baseline state of the brain.

Nodes within the DMN may serve distinct functions, although the relevance of each area to the overall network may lie on a continuum. For example, using a data-driven partial correlation analysis, Fransson et al. show that only (posterior cingulate cortex / precuneus are correlated with all other DMN areas [27]. Additionally, Uddin et al. have found that different nodes of the default mode demonstrated differential functional connectivity to modulate separate task-positive networks [66]. That is, the posterior cingulate cortex negatively predicted activity in prefrontal motor control areas, while the ventromedial prefrontal cortex was negatively correlated with medial parietal attention networks. The posterior cingulate cortex has independently been implicated in self-referential processes, such as prospection, recollection, and theory of mind [16] and responds to physical characteristics of visual stimuli such as texture, size, and brightness independent of whether attention is paid to stimuli [70]. Activity in the posterior cingulate cortex / precuneus has also been

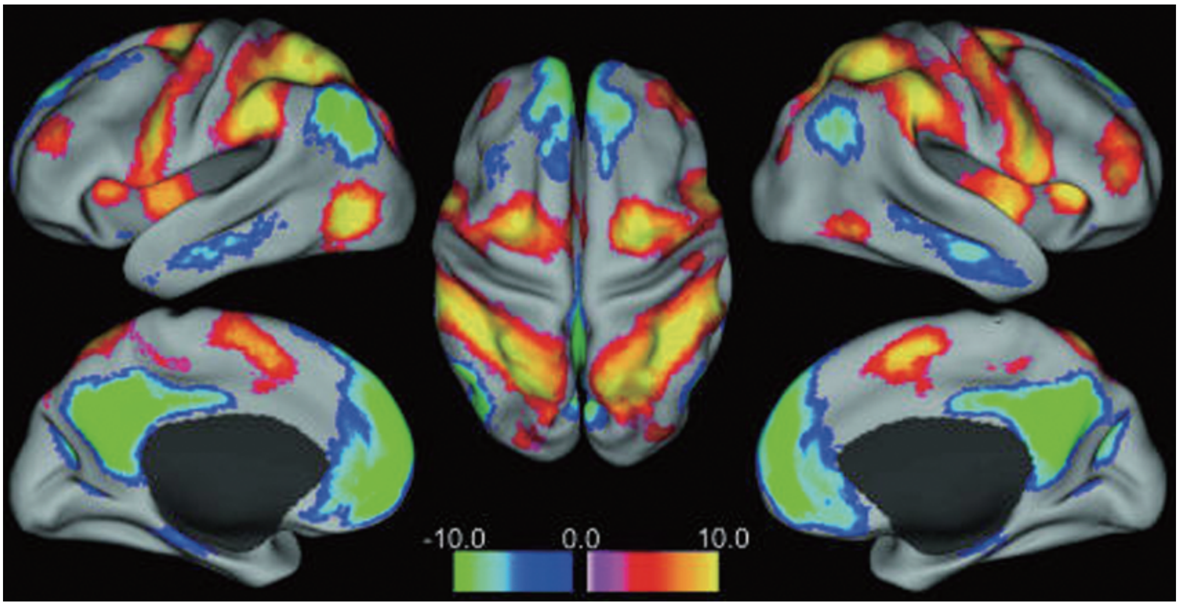

Fig. 1. Composite correlation map showing two anti-correlated networks. The posterior cingulate / precuneus, lateral parietal cortex (LP), and medial prefrontal cortex (MPF) are positively correlated with each other and are consistent with the default mode network. Regions negatively correlated with the default mode network include the intraparietal sulcus (IPS), the frontal eye fields (FEF), and the middle temporal region (MT+). These regions have been implicated in attention-demanding cognitive tasks. Reproduced with permission from Fox et al., *PNAS*, 2005.

associated with retrieval of episodic memories [45,46]. In contrast, the ventromedial prefrontal cortex has been linked to emotional and social processing [52], and may be responsible for integrating emotional biases in decision making processes [2]. In addition the medial and orbital frontal cortices are involved in assessing emotional salience of stimuli [27]. Finally, the lateral parietal cortex may play a role in semantic processing and attention [6]. Paradoxically, it seems that while these regions are suppressed during task-directed activity, many also serve essential roles in monitoring sensory input.

The default mode network has been consistently found in normal subjects during rest using a data-driven, independent component analysis of functional magnetic resonance imaging (fMRI) data [50]. Disruption of the DMN has been associated with many conditions in which various aspects of cognitive function are reduced, including reduced connectivity in normally developing children [65] as well as disorders such as autism [36], ADHD [17], Alzheimer's disease [30], schizophrenia [72], and depression [31].

Furthermore, insights from studies of the DMN on altered states of consciousness have been particularly revealing, and impairment of frontoparietal areas associated with the default mode has been implicated in other states of altered consciousness. For example, PET and fMRI studies have demonstrated that unconsciousness in the vegetative state is associated with decreases in connectivity between the thalamus and the anterior cingulate; and that connectivity increases when patients recover consciousness [42]. Furthermore, decreased metabolism in the prefrontal and posterior cingulate cortex / precuneus areas and decreased connectivity between these regions have been demonstrated in vegetative patients, with restored metabolism in the precuneus after clinical recovery [40,41]. In contrast, function in arousal structures including the reticular formation, hypothalamus, and the basal forebrain remain relatively intact in the vegetative state [43]. BOLD signal decreases [19] and decreased glucose metabolism [47] have also been measured in default mode areas in non-REM sleep. Finally, anesthetic agents have been demonstrated to decrease cerebral blood flow to the precuneus in a dose dependent manner [35]. In this review, we present evidence that, similarly to these other states of loss of consciousness, epilepsy affects specific default mode areas in causing impaired consciousness.

## 3. Default mode network involvement in complex partial seizures

Although complex partial seizures are often caused by temporal lobe pathology and have foci limited to one hemisphere, these events can still cause loss of consciousness [34]. While ictal and post-ictal amnesia might be expected in a disease originating from medial temporal lobe structures important for memory, it is less clear why focal seizures in the temporal lobe would affect consciousness without generalizing to the rest of the brain. Several hypotheses exist as to why temporal lobe epilepsy could cause loss of consciousness. It is possible that ictal hyperperfusion of the temporal lobe causes shunting of blood away from other areas of the brain responsible for maintenance of consciousness, though this is unlikely given that the medial temporal lobe and areas of higher order function such as the fronto-parietal cortex have separate vascular supplies. A more likely possibility is that widespread decreases in cerebral function during complex partial seizures have a primarily neural mechanism. We would like to propose a hypothesis in which complex partial seizures cause loss of consciousness by selective inhibition of subcortical arousal systems leading to depressed function of the higher order association cortex, including default mode network areas.

A confluence of evidence from multiple techniques has implicated areas of the default mode in complex partial seizures, with a preponderance of evidence for temporal lobe epilepsy. For example, neuroimaging studies in humans have shown decreased cerebral blood flow (CBF) in frontal and parietal cortex during these events [18,51,59] as well as with interictal discharges [39] causing deactivation in bilateral posterior cingulate during interictal spikes [37].

Using single photon emission computed tomography (SPECT), Van Paesschen and colleagues found ictal hypoperfusion in the superior frontal gyrus and precuneus in 90% of temporal lobe epilepsy patients [67]. We performed a simultaneous video-EEG and SPECT study of patients with confirmed mesial temporal sclerosis to investigate patterns of cerebral blood flow during seizures with or without impaired consciousness [10]. Interestingly, focal decreases were observed in DMN regions such as the posterior cingulate cortex / precuneus, inferior parietal lobule, and medial frontal/anterior cingulate cortex, as well as in orbital frontal, and inferior lateral frontal cortex during complex partial seizures with decreased consciousness. These changes, however, were not seen during simple partial seizures with spared consciousness (Fig. 2). Furthermore, primary sensory cortices were spared even with loss of consciousness. These results were confirmed with studies using intracranial EEG recordings of temporal lobe seizures to directly measure neuronal activity using the

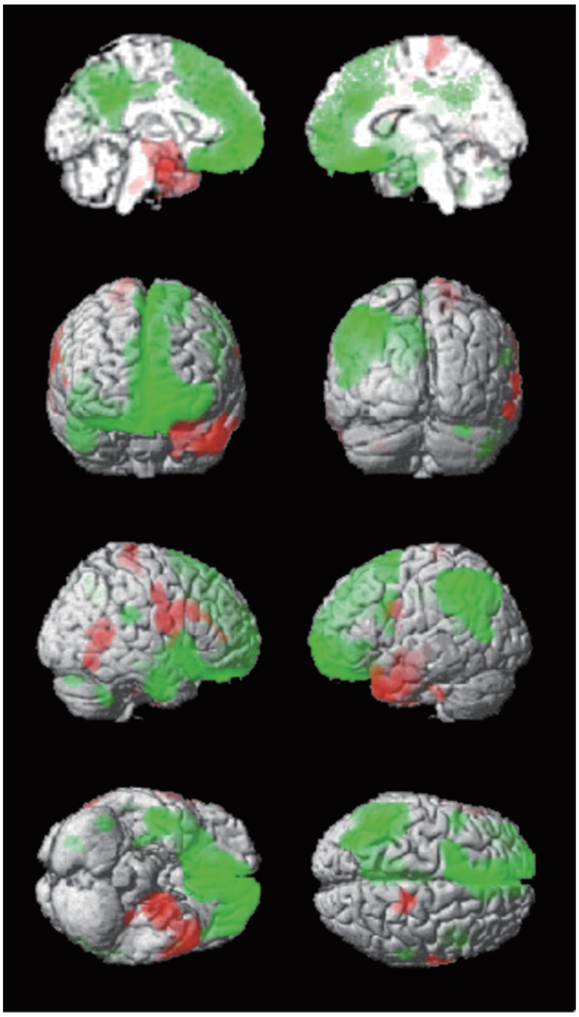

Fig. 2. Ictal cerebral blood flow increases (red) and decreases (green) 60–90 s after onset of complex partial temporal lobe seizures. Analysis of complex partial seizures from eight patients was performed with height threshold $p < 0.01$ and extent threshold $k = 125$ voxels. Note the marked increases in CBF in the temporal lobe on the side of seizure onset, and the widespread decreases occurring in fronto-parietal association cortices including the default mode network. Reproduced with permission from Blumenfeld et al., Cerebral Cortex, 2004.

same patient population, anatomic regions, and time intervals. We observed a slowing of intracranial EEG activity in frontoparietal association areas during and immediately after seizures, suggesting impaired cortical function in these areas, with relative sparing of primary sensory areas [11]. Together, these findings show that reduced CBF in default mode areas in temporal lobe seizures is related to a profound change in the physiology of these regions including cortical slow-wave oscillations resembling sleep, coma or encephalopathy. It is therefore unlikely that decreased CBF in the DMN during temporal lobe seizures is simply due to "suspension" of DMN activity as has been proposed during normal cognitive tasks. Furthermore, we found that reduced activity in DMN regions was correlated with increased CBF in the medial thalamus, suggesting a possible subcortical mechanism for these cortical changes [10].

The recent development of an animal model has shed additional light on the mechanisms by which focal temporal lobe seizures cause loss of consciousness via long-range disruption by subcortical activating systems of neocortex [23–25]. In particular, fMRI decreases were found in the orbital frontal, anterior cingulate, and posterior cingulate cortex / retrosplenial cortex during seizures similar to changes observed in human patients. Furthermore, while electrophysiological recordings from the hippocampus demonstrated fast 9–12 Hz fast polyspike seizure activity, the frontal cortex exhibited 1–3 Hz slow waves resembling slow-wave sleep oscillations, decreased mean rate of neuronal firing, and decreased neuronal metabolism associated with arrest of spontaneous exploratory behaviors. In the same animal model, we found that connections via the fornix between the hippocampus and subcortical structures appear to be critical for generating ictal neocortical slow activity and the associated behavioral arrest [25].

Based on these combined findings from human patients and an animal model, we have proposed a "network inhibition hypothesis" by which seizures cause impaired consciousness by affecting entire networks rather than a neural locus; and widespread cortical deactivation by inhibiting the subcortical activating system (Fig. 3) [8,13,57]. For temporal lobe epilepsy, it seems that loss of consciousness occurs by seizure activity arising from the temporal lobe and propagating to subcortical structures; this may inhibit the arousal functions of the thalamus, basal forebrain and upper brainstem, to widely deactivate frontal and parietal association cortex [24,71]. In contrast, temporal lobe seizures that do not cause loss of consciousness may only produce impaired activity at the seizure foci without widespread effects via propagation to midline subcortical structures, and would thus spare cortical regions including the DMN.

## 4. Default mode network involvement in generalized tonic-clonic seizures

Generalized tonic-clonic seizures are perhaps the most well known and most dangerous seizure type and are marked by large-scale increases in neuronal firing, muscle contractions, autonomic and neuroendocrine changes, and lapses in consciousness. Generalized tonic-clonic seizures are associated with primary generalized epilepsy and can also occur when partial seizures spread across the cortex and secondarily generalize.

There is increasing evidence that so-called generalized seizures are in fact of focal origin and that even during generalization some networks are involved more intensely that others [9,20,49,62]. Due to the motor involvement during tonic-clonic seizures, however, imaging studies of the specific networks involved cannot be performed in human patients using fMRI or PET imaging. However, ictal single photon emission computed tomography (SPECT) works by injection of radiopharmaceutical during the seizure; and imaging at a later time can provide a window into changes in cerebral blood flow during seizures, a known marker of neuronal activity. Additionally, intracranial electroencephalography (EEG) [62] and electroconvulsive therapy (ECT) studies have proven useful for understanding these events [7,9,21].

Functional neuroimaging in animal models of generalized tonic-clonic seizures has shown increases in neuronal activity in both cortical and subcortical networks which are widespread and bilateral [20,56,63] but selectively involve specific regions of cortex most intensely [20]. Similar increases have been captured in humans as well. SPECT studies have revealed that the earliest increases are often in the temporal lobe, where tonic-clonic seizures most commonly originate [14,68]. Additionally, large CBF increases during the generalization phase have been observed in the bilateral medial and lateral parietal cortex [9,21] as well as in the thalamus, basal ganglia, and superior medial cerebellum [9,14,21]. Interestingly, we recently observed that during the post-ictal phase, CBF in the bilateral lateral cerebellar hemispheres continues to increase before returning to a baseline level [14]. These findings are in line with an earlier study in a cat model of generalized tonic-clonic seizures showing increased cerebellar Purkinje cell firing in the late ictal and post-ictal phases [61]. Since Purkinje cells send inhibitory outputs to influence thalamocortical circuits, it was postulated that increased cerebellar activity may contribute to seizure termination and / or to post-ictal suppression.

In addition to the focal increases observed during generalized tonic-clonic seizures, CBF hypoperfusion has also been reported in many DMN areas of the brain. SPECT studies of ECT-induced seizures in depression

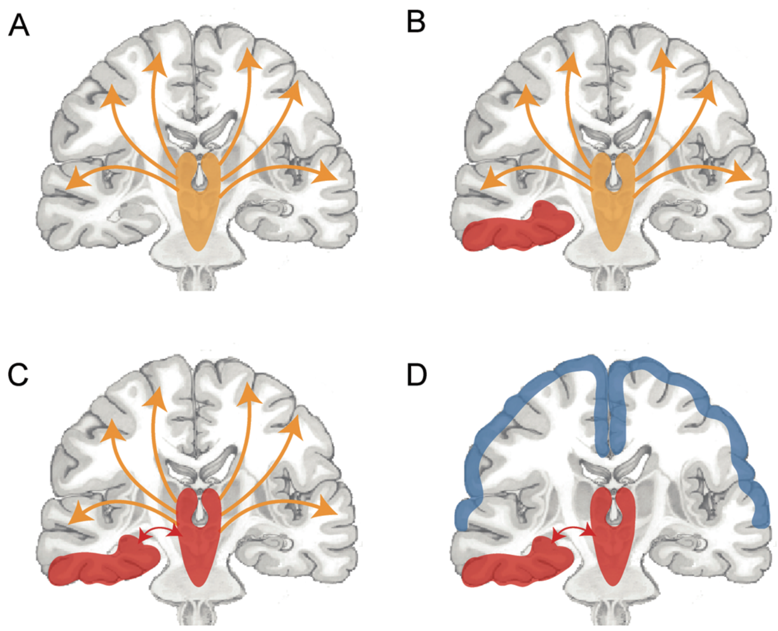

Fig. 3. Network inhibition hypothesis. A. Interaction between upper brainstem and thalamus with cerebral cortex to maintain consciousness under normal conditions. B. Temporal lobe focal seizure without secondarily generalization. C. Propagation of seizure activity to midline subcortical structures. D. Resulting suppression of activity in fronto-parietal cortices leading to loss of consciousness. Reproduced with permission from Blumenfeld and Taylor, *The Neuroscientist*, 2003.

patients have revealed significant CBF decreases in the bilateral anterior and posterior cingulate gyrus and precuneus [7,21] in addition to decreases in the medial frontal and lateral parietal lobes [9]. These findings were largely recapitulated in a subsequent study of a large group of epilepsy patients, which reported ictal and post-ictal CBF decreases in the precuneus and cingulate gyrus and postictal CBF decreases in the lateral frontoparietal association cortex [14].

Of note, the degree of ictal and postictal cerebellar CBF increases was strongly correlated with CBF *increases* in the thalamus and midbrain, and with CBF *decreases* in frontoparietal default mode regions (Fig. 4) [14]. Given that the cerebellum primarily consists of inhibitory purkinje cells, the positive correlation between the cerebellum and thalamus may represent active inhibition of thalamocortical networks, resulting in the negative correlation seen between the cerebellum and DMN regions. Although the precise changes leading to thalamocortical inhibition differ from those seen during complex partial seizures, the general mechanism at work still generally supports a "network inhibition hypothesis" in which increased activity in some regions leads to widespread decreased cortical activity via subcortical inhibitory mechanisms.

## 5. Default mode network involvement in absence seizures

Absence seizures, common in children with childhood absence epilepsy and in adults with idiopathic generalized epilepsy, are non-convulsive events that often cause brief disruptions in attention or consciousness. They are accompanied by a well-characterized bilaterally synchronous 3–4 Hz spike-and-wave discharge (SWD) pattern on EEG. Despite the stereotyped electrical pattern seen in absence seizures, the associated degree of behavioral impairment can vary widely across patients and even across seizures within the same patient. On a continuous performance task, for example, omission rates during seizures in children with absence epilepsy are 81%, while omission rates on a simpler repetitive tapping task are only 39% [5].

This variability in attentional impairment is likely related to the variable focal disruption of specific networks during these so-called "generalized" events. Early EEG and PET studies [22] suggested that absence seizures are whole brain events without focal origin, though more recent fMRI studies have shed light on a more complex mechanism of seizure initiation and propagation. FMRI studies in the WAG/Rij and GAERS rat models of absence seizures have demonstrated robust focal BOLD signal increases in the perio-

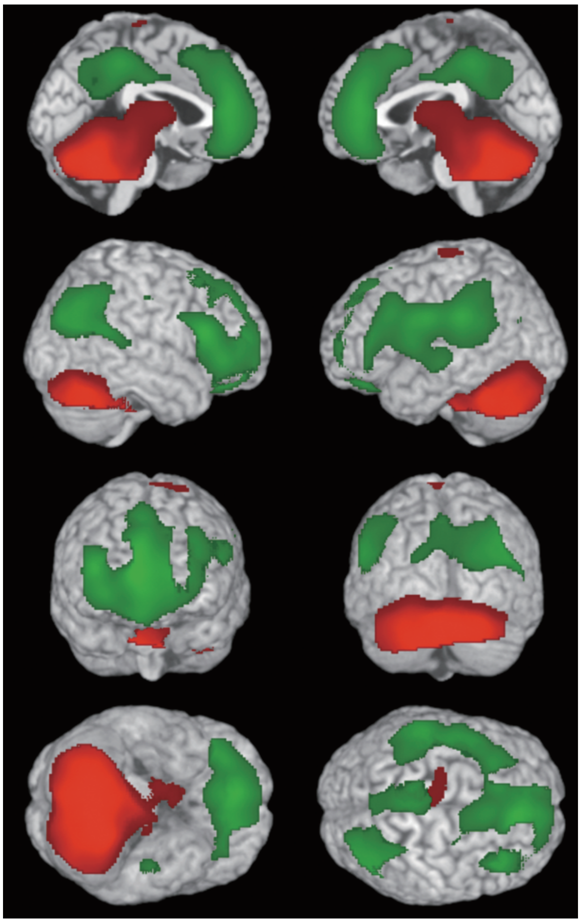

Fig. 4. Generalized tonic-clonic seizures: network correlations between CBF in cerebellum and other regions of the brain, including many default mode structures. Analysis of ictal and postictal CBF in 59 secondarily generalized tonic-clonic seizures was performed in SPM at height threshold $p < 0.01$, and both positive (red) and negative (green) correlations are shown. CBF changes in the upper brainstem and thalamus were significantly positively correlated with cerebellar CBF changes. Significant negative correlations were seen in central nodes of the DMN, including the bilateral fronto-parietal association cortex, anterior and posterior cingulate, and the precuneus. Reproduced with permission from Blumenfeld *et al.*, *Brain*, 2009.

ral somatosensory cortex [32,56], and electrophysiology studies in the same animal model have shown maximal local field potential activity in the same region [49, 55].

Many of the BOLD signal changes seen in these animal models have been replicated in humans as well. Aghakhani et al [1] were able to capture robust thalamic involvement during SWD in idiopathic generalized epilepsy using 1.5T fMRI, and Laufs et al. [39] reported posterior thalamic activation at 3T. Robust thalamic involvement has also been reported during seizures in childhood absence epilepsy [4,5,53]. Other areas of activation include midfrontal regions [5,28] and the insulae [28].

In addition to the corticothalamic BOLD signal increases often reported during SWD, absence seizures are also associated with well-characterized decreases in activity in brain regions, many of which coincide with the default mode network (Fig. 5). Most decreases during absence seizures occur >7 s after electrographic seizure onset [4,28]. Archer et al. [3] reported significant SWD-related deactivation of the posterior cingulate, and BOLD signal decreases have also been reported in the parietal cortex [5,28,53], precuneus [53], caudate nucleus [53], cingulate gyrus [5,28], basal ganglia [5], and posterior temporal cortices [28]. CBF measurements have also confirmed that decreases occur in the same regions showing negative BOLD responses [33].

Unlike other seizure types, however, absence seizures seem to involve DMN structures even before electrographic seizure onset. A recent timecourse analysis [4] revealed that significant BOLD signal increases occur in the orbital frontal, cingulate gyrus, lateral parietal, and precuneus areas more than five seconds before initial EEG changes. That many of these same regions subsequently show typical DMN decreases (Fig. 5) suggests that the fundamental mechanism of DMN decreases during these events might represent an undershoot phenomenon as these neurons recover from the early abnormal activation. Interestingly, however, thalamic fMRI increases also occur relatively late and roughly correspond to the time of widespread cortical fMRI decreases [4], again suggesting that cortical-subcortical interactions may play a role in the observed decreases in the DMN.

Thus, these observations may support the network inhibition hypothesis during absence seizures as well. Gotman et al. [28] have also investigated the timing of the deactivations seen during SWD. Whereas most positive responses are seen relatively early, the negative responses tended to peak approximately 7–9 seconds following SWD onset [28]. One possible explanation for this phenomenon is suspension of the DMN [28], although further work is needed to determine the mechanisms for these changes.

## 6. Future directions and conclusions

Elucidation of the default mode network originally arose from studies of healthy subjects; however, it seems clear that this network plays a central role in the

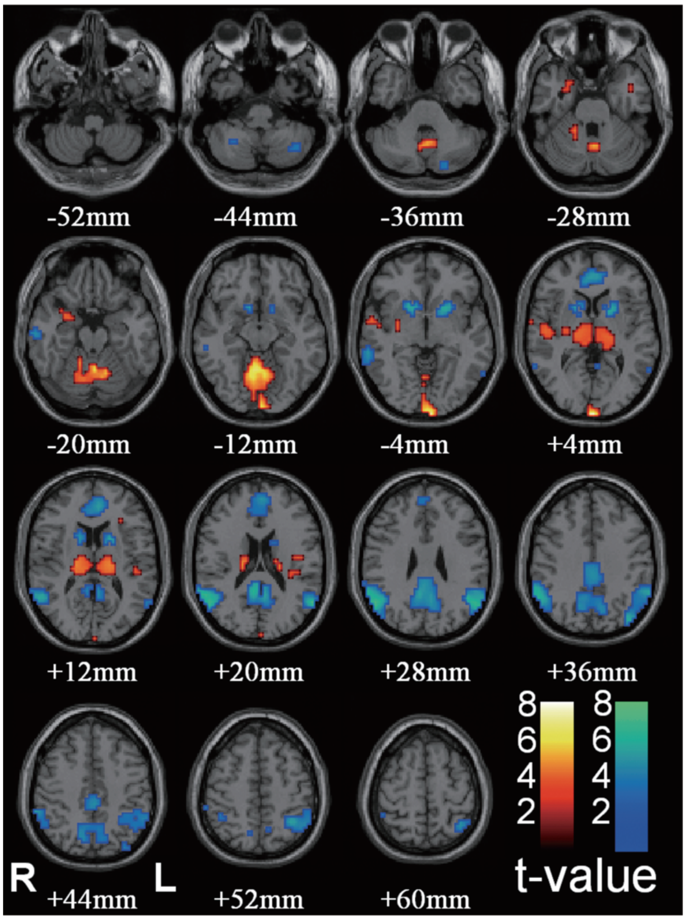

Fig. 5. FMRI BOLD signal increases (red) and decreases (blue) during 54 absence seizures in 9 patients. The analysis was performed in SPM2 using the general linear model approach and the conventional haemodynamic response function. This analysis revealed large significant bilateral increases in the bilateral thalamus, among other regions. In addition, significant decreases were observed in DMN regions, including the bilateral lateral parietal cortex, the medial parietal and medial frontal cortex, as well as in the basal ganglia. These findings provide in a sense an "ictal snapshot", as further analysis of the BOLD signal timecourse surrounding these seizures revealed a complex series of changes not detectable with conventional HRF modeling. Reproduced with permission from Bai et al., *J. Neurosci.*, 2010.

clinical manifestations of seizure activity as well. In all three major types of seizures in which patients experience loss of consciousness (complex partial, tonic-clonic, and absence), there is well-documented evidence of focal decreased activity in the DMN. Moreover, the thalamus is strongly involved in all three seizure types, suggesting corticothalamic networks are at play.

This confluence of evidence, confirmed by a wide range of neuroimaging modalities, suggests that a common mechanism may participate in the suppression of DMN activity in these diverse seizure types. One possibility is that, like in normal brain function (Fig. 1) [26] a "push-pull" relationship may exist between activation of certain brain networks and deactivation of the DMN. Thus, activation of particular brain regions either by normal activity or by seizures may lead to decreased activity in the DMN through mechanisms yet to be determined. Based on human and animal studies of complex partial temporal lobe seizures, we have proposed a network inhibition hypothesis by which increased cortical activity in one region inhibits subcortical arousal systems, leading to widespread decreased cortical activity including the DMN (Fig. 3). It is unclear whether similar or analogous mechanisms may also play a role in "generalized" tonic-clonic and absence seizures. However, the time-varying nature of the signals may also be a crucial factor in determining changes in the DMN. For example, in both generalized tonic-clonic and absence seizures, *increases* are initially seen in DMN cortical regions, followed later by profound and long-lasting *decreases* in DMN network activity. These cortical decreases are accompanied by subcortical increases, for example in the thalamus and cerebellum, raising the possibility that the DMN decreases may not simply represent "undershoot" following the earlier increases, but could involve active subcortical inhibitory processes. The detailed mechanisms and implications of these network changes for understanding both normal and abnormal brain function will require further investigation.

## Acknowledgements

This work was supported by NIH R01 NS055829, R01 NS049307, P30 NS052519, The Donaghue Foundation, the Betsy and Jonathan Blattmachr Family (HB), and by NIH MSTP TG 2T32GM07205 (JG).
